# False-positive results for pheochromocytoma associated with norepinephrine reuptake blockade

**DOI:** 10.1530/ERC-23-0063

**Published:** 2023-12-01

**Authors:** Robin Schürfeld, Christina Pamporaki, Mirko Peitzsch, Nada Rayes, Osama Sabri, Silvio Rohm, Ronald Biemann, Benjamin Sandner, Anke Tönjes, Graeme Eisenhofer

**Affiliations:** 1 Division of Endocrinology and Diabetes, Department of Internal Medicine, University of Leipzig, Leipzig, Germany; 2 TU Dresden, Medical Clinic III, University Hospital Carl Gustav Carus, Dresden, Germany; 3 TU Dresden, Institute of Clinical Chemistry and Laboratory Medicine, Dresden, Germany; 4 Center of Surgery, Division of Endocrine Surgery, Department for Visceral, Transplant, Thoracic, and Vascular Surgery, University of Leipzig, Leipzig, Germany; 5 Department of Nuclear Medicine, University of Leipzig, Leipzig, Germany; 6 Center of Surgery, Department for Visceral, Transplant, Thoracic, and Vascular Surgery, University of Leipzig, Leipzig, Germany; 7 Center of Surgery, Department for Vascular Surgery, Diakonissen Hospital of Leipzig, Leipzig, Germany; 8 Institute of Clinical Chemistry and Laboratory Medicine, University of Leipzig, Leipzig, Germany

**Keywords:** pheochromocytoma, false-positive, doxepin, duloxetine, metanephrines, normetanephrine, tricyclic antidepressant, serotonin–norepinephrine reuptake inhibitor, metanephrine, methoxytyramine, clonidine

## Abstract

Measurements of plasma metanephrines and methoxytyramine provide a sensitive test for diagnosis of pheochromocytoma/paraganglioma. False-positive results remain a problem, particularly in patients taking norepinephrine reuptake-blocking drugs. Therefore, in this retrospective observational study, we measured plasma metanephrines and methoxytyramine in 61 patients taking norepinephrine reuptake blockers (tricyclic antidepressants or serotonin–norepinephrine reuptake inhibitors) and 17 others taking selective serotonin reuptake inhibitors, all without pheochromocytoma/paraganglioma. We highlight a singular case with strongly elevated plasma normetanephrine and methoxytyramine concentrations associated with norepinephrine reuptake blockade. Data were compared to results from 252 and 1804 respective patients with and without tumors. Plasma normetanephrine was 40% higher (*P* < 0.0001) in patients on norepinephrine reuptake blockers and methoxytyramine was 127% higher (*P* = 0.0062) in patients taking tricyclic antidepressants compared to patients not taking uptake blockers and without tumors. The corresponding false-positive rates rose (*P* < 0.0001) from 4.8% to 23.0% for normetanephrine and from 0.9% to 28.6% for methoxytyramine. Selective serotonin reuptake inhibitors did not increase plasma concentrations of metabolites. In the highlighted case, plasma normetanephrine and methoxytyramine were elevated more than six times above upper reference limits. A pheochromocytoma/paraganglioma, however, was excluded by functional imaging. All biochemical test results normalized after discontinuation of norepinephrine reuptake blockers. These findings clarify that norepinephrine reuptake blockers usually result in mild elevations of normetanephrine and methoxytyramine that, nevertheless, significantly increase the number of false-positive results. There can, however, be exceptions where increases in normetanephrine and methoxytyramine reach pathological levels. Such exceptions may reflect failure of centrally mediated sympathoinhibition that normally occurs with the norepinephrine reuptake blockade.

## Introduction

Pheochromocytomas and paragangliomas (PPGL) are neuroendocrine tumors derived from chromaffin cells of the adrenal medulla or extraparaganglionic tissue (Tischler 2008). Measurements of plasma-free normetanephrine and metanephrine provide high sensitivity for the diagnosis of PPGL (Lenders *et al.* 2002), and even more so when combined with additional measurements of the *O*-methylated dopamine metabolite methoxytyramine (Eisenhofer *et al.* 2018). However, low prevalence of these tumors in daily clinical practice combined with suboptimal specificity means that false-positive results considerably outnumber true-positive results (Yu & Wei 2010).

When blood samples for plasma-free metanephrines are collected under optthree groups of patientsimized conditions (e.g. supine position and overnight fast), the false-positive rate is around 5% and can increase to over 25% when those conditions are not followed (Eisenhofer *et al.* 2003, Darr *et al.* 2014). Among the causes of false-positive results, drug interferences are responsible for around 20% of all false-positive results (Yu & Wei 2010). Drugs that block norepinephrine reuptake, such as tricyclic antidepressants (TCAs), are in particular well established to increase rates of false-positive results for plasma and urinary normetanephrine (Eisenhofer *et al.* 2003). Phenoxybenzamine, well-known as a nonselective alpha-adrenoceptor blocker, has also been documented to block norepinephrine uptake (Iversen & Langer 1969). In one study, TCAs and phenoxybenzamine accounted for 41–45% of all elevated levels of norepinephrine and normetanephrine in patients without PPGL (Eisenhofer *et al.* 2003). Other norepinephrine reuptake-blocking drugs include serotonin–norepinephrine reuptake inhibitors (SNRIs), which in one case report were responsible for a large increase in plasma nometanephrine (Neary *et al.* 2011). Although it was shown 20 years earlier that selective serotonin reuptake inhibitors (SSRIs) do not cause elevations or false-positive test results for plasma metanephrines (Eisenhofer *et al.* 2003), it remains unclear whether the new generation of SSRIs now widely used may be a problem.

The aim of this retrospective observational study was to further clarify increases in plasma concentrations and false-positive results for free normetanephrine, metanephrine and methoxytyramine due to norepinephrine reuptake inhibitors (NRIs), including TCAs and SNRIs, and SSRIs. For this purpose, plasma concentrations of these metabolites in patients taking NRIs or SSRIs were examined and compared to plasma concentrations in patients with and without PPGLs. To further characterize the effects of NRIs on norepinephrine reuptake, we examined plasma concentrations of norepinephrine and its intraneuronal metabolite dihydroxyphenylglycol (DHPG), which serves as a marker of norepinephrine reuptake (Goldstein *et al.* 1988). We also outline findings in an exceptionally illustrative patient who presented with elevations of plasma normetanephrine and methoxytyramine more than six-fold above the upper cutoffs of reference intervals yet did not have a PPGL. To clarify the unusual nature of this presentation, we compared the results of this patient to those of the patients with and without PPGL.

## Methods

### Study populations

The focal data for this retrospective observational study are from 79 patients taking inhibitors of norepinephrine and/or serotonin uptake. These patients included 41 taking TCAs, 20 taking SNRIs and 17 taking SSRIs (Table 1). Among the 79 patients, this report also focuses on an additional 74-year-old female taking both a TCA and a SNRI. This selected exceptional patient was worked up according to the 2013 CARE guidelines checklist (Riley *et al.* 2017).

**Table 1:** Patient characteristics according to the three groups taking uptake blockers and the two comparison groups of patients with and without PPGL.

	Patients on uptake blockers			Comparison groups	
	TCA	SNRI	SSRI	No PPGL	PPGL
*n*	41	20	17	1804	252
Sex (female/male)	25/16	15/5	11/6	897/907	144/108
Age	53 (41–62)	56 (45–68)	59 (56–63)	53 (42–63)	50 (40–61)

Values for age are shown as medians with interquartiles. Treatment with TCAs included 29 patients on amitriptyline, 3 patients each on imipramine and nortriptyline, 2 patients each on doxepin and desipramine and 1 patient on trimipramine. One other patient was taking maprotiline. Among the 20 patients taking SNRIs, 14 were on venlafaxine and 6 were on duloxetine. Among the 17 patients taking SSRIs, 8 were on escitalopram, 5 were on citalopram, 2 were on fluoxetine, 1 was on paroxetine and 1 was on sertraline. PPGL, pheochromocytoma and paraganglioma; SNRI, serotonin–norepinephrine reuptake inhibitor; SSRI, selective serotonin reuptake inhibitor; TCA, tricyclic antidepressant.

Among the 79 patients taking uptake-blocking drugs, 12 were recruited at Leipzig, including the exceptional female patient taking both a TCA and a SNRI. While for the exceptional patient written informed consent of the guardian was obtained, the other 11 patients provided broad informed consent within the hospital admission processes with approval of the Data Protection Authorities of German federal states at the University Hospital of Leipzig (Art. 9 Abs. 2 lit. i i.V.m. Art. 89 Abs. 1 DS-GVO u. § 34 SächsKHG). The other 67 subjects were recruited into one or more of three intramural review board-approved clinical protocols: (i) the Prospective Monoamine-producing Tumor study (PMT, EK 189062010); (ii) the PROspective study on the diagnostic value of Steroid profiling in primary ALDOsteronism (PROSALDO, EK 386102018) and (iii) the registry and biobank of the European Network for the Study of Adrenal Tumors (ENS@T, EK 23012014). The two former protocols provide for prospective recruitment of patients with suspected PPGLs or primary aldosteronism, whereas the latter serves as a retrospective study registry that allows for recruitment of patients with adrenal tumors, including those enrolled into other study protocols. All patients and volunteers enrolled via clinical protocols provided a written informed consent for their participation.

For comparisons to the focal data derived from patients on uptake blockers, additional data were included from 1804 and 252 respective patients in whom PPGLs were excluded and confirmed (Table 1). These included 236 with PPGLs taken from a publicly available dataset (Eisenhofer *et al.* 2018) and a further 16 patients thereafter identified with PPGL and also recruited via the Dresden-based PMT study (Table 1). In the initial PMT study, patients taking TCAs were excluded, while those taking SNRIs and SSRIs were not. Thus, 16 of the original 1820 patients without PPGLs in the publicly available dataset of the PMT study were identified to be taking SNRIs or SSRIs; those patients were hence excluded from that group, leading to a total of 1804 patients without tumors.

For further comparisons involving plasma DHPG and norepinephrine, advantage was taken of an additional dataset from 603 hypertensive and normotensive volunteers (334 females, median age 41 years). These volunteers were originally recruited for purposes of establishing reference intervals for different biomarkers, including plasma metanephrines and methoxytyramine as detailed elsewhere (Eisenhofer *et al.* 2019).

### Confirmation and exclusion of PPGLs

PPGLs were confirmed either by pathological examination of surgically resected tumors or in isolated cases by functional imaging of disease (e.g. if a surgical resection was not possible due to metastases or rejection of surgery). Exclusion of PPGL was largely based on patient follow-up but also included negative results of imaging studies or alternative diagnoses as extensively detailed elsewhere (Eisenhofer *et al.* 2018).

### Biochemical analysis

Patients underwent an overnight fast and maintained a supine position for 30 min until blood samples were drawn into heparinized tubes, which were placed on ice or cold packs before centrifugation. Plasma specimens were either immediately processed or stored at −80^o^C, before laboratory analyses were performed, all in the same laboratory. Plasma concentrations of free normetanephrine, metanephrine and methoxytyramine were measured by liquid chromatography with tandem mass spectrometry after 2013 or, prior to 2013, by liquid chromatography with electrochemical detection (LC-ECD) as previously described (Peitzsch *et al.* 2013). Age-specific reference intervals for plasma normetanephrine were used as outlined elsewhere (Eisenhofer *et al.* 2013). Plasma catechols, including norepinephrine, epinephrine, dopamine, DHPG, dihydroxyphenylalanine and dihydroxyphenylacetic acid were measured by LC-ECD (Eisenhofer *et al.* 1986).

### Data analysis and statistical analysis

Based on previous findings (Esler *et al.* 1991), the underlying hypothesis behind the present analysis was that variable increases in plasma concentrations and false-positive results for plasma normetanephrine in patients taking NRIs reflect the balance between actions of these drugs to centrally induce sympathoinhibition and peripherally block norepinephrine reuptake in sympathetic nerves. To explore this hypothesis, we evaluate exceptionally high plasma concentrations of normetanephrine and methoxytyramine in an illustrative patient taking NRIs and compare results in three groups of patients taking TCAs, SNRIs or SSRIs to those in larger groups of patients with and without PPGL.

Differences between groups for continuous parameters were determined by the Steel–Dwass nonparametric method for multiple comparisons. Due to the large size of the population tested for PPGL who were not taking uptake blockers and who did not have tumors, examination of differences was primarily directed to this group compared to the three different groups of patients taking TCA, SNRIs or SSRIs. If differences in plasma concentrations were observed, rates of false positives were compared for the groups showing those differences. Differences in false-positive rates between those groups were determined by the chi-square test for noncontinuous parameters.

Differences in plasma norepinephrine and DHPG concentrations in the presence and absence of NRIs were evaluated using the nonparametric Mann–Whitney *U* test. The significance of relationships was established using the Spearman’s rank correlation coefficient (*r*_s_). All statistical analyses were performed using the JMP Pro statistical software package, version 15. Values in tables, graphs and text are shown as medians with interquartile ranges.

## Results

### Plasma normetanephrine, metanephrine and methoxytyramine

**Figure 1:**
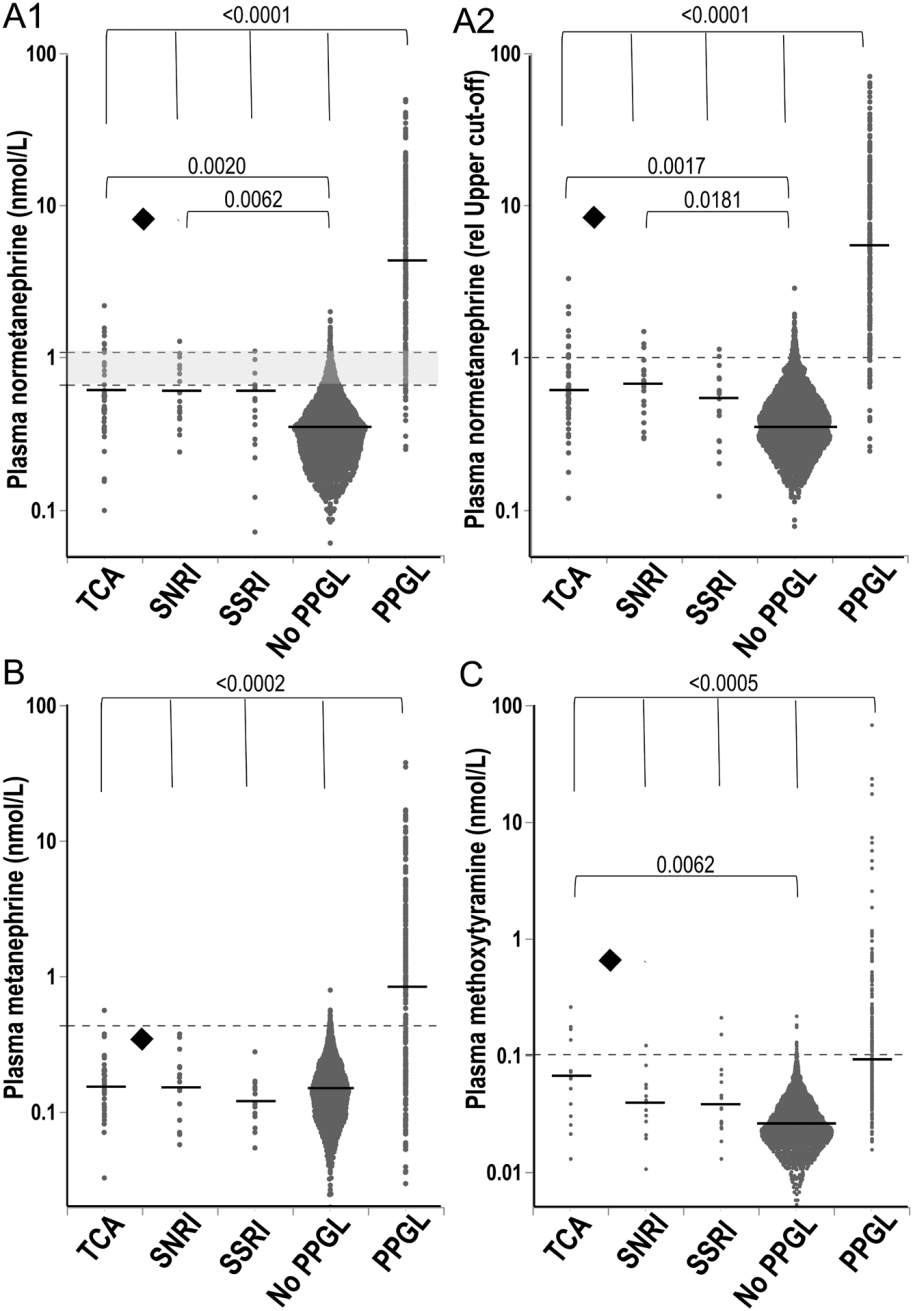
Dot plot display of plasma normetanephrine (A), metanephrine (B) and methoxytyramine (C) concentrations for patients taking tricyclic antidepressants (TCAs), mixed serotonin norepinephrine reuptake inhibitors (SNRIs) and selective serotonin reuptake inhibitors (SSRIs) compared to patients without and with pheochromocytoma or paraganglioma (PPGL). There were missing data for methoxytyramine in 24 patients without PPGL, in 11 patients without PPGL taking NRIs and in one patient with PPGL. For metanephrine, there were missing data in one patient taking SNRI. The black highlighted diamond indicates the patient for the case presentation who was taking both a TCA and an SNRI. Dashed horizontal lines with the gray area in panel A1 illustrate age-specific upper cutoffs for plasma normetanephrine with highest cutoff value of 1.04 nmol/L for patients >60 years. Figure A2 shows the plasma normetanephrine concentration in relation to the age-specifics upper cutoffs, (i.e. values above 1 indicate elevated normetanephrine levels). Dashed horizontal lines in B and C show upper cutoff limits for plasma metanephrine or methoxytyramine. Horizontal black lines show medians for each group. Differences between groups are shown as *P*-values by the Steel–Dwass nonparametric method for multiple comparisons. *Y*-axes are shown as logarithmic scales.

Plasma concentrations of normetanephrine in patients taking TCAs and SNRIs were 42% and 35% higher (*P* < 0.01), respectively, than in the patient group without PPGLs but showed no difference for patients taking SSRIs (Fig. 1A). Corresponding rates of false-positive results were 4.8-fold higher in the combined group of patients taking TCAs and SNRIs than in the patient group without PPGLs (23.0% vs 4.8%, *P* < 0.0001).

In contrast to normetanephrine, plasma concentrations of metanephrine and corresponding rates of false positives were not higher among any group of patients taking uptake blockers compared to the patient group without PPGL (Fig. 1B).

There were no differences in plasma concentrations of methoxytyramine among patients taking SNRIs or SSRIs and the group of patients without PPGLs, whereas patients taking TCAs showed 127% higher (*P* = 0.0062) plasma concentrations of methoxytyramine than those without PPGL (Fig. 1C). The corresponding rates of false-positive test results for methoxytyramine were thus higher in the patients taking TCAs than in the group of patients without PPGL (28.6% vs 0.9%, *P* < 0.0001).

No differences in plasma concentrations and rates of false positives for normetanephrine and methoxytyramine were observed between the three groups of patients on TCA, SNRIs and SSRIs (Fig. 1A and C). However, relatively small sizes of these three groups (*n* = 41, 20 and 17) compared to the larger size (*n* = 1804) of the comparison group without PPGL and who were not taking uptake blockers offered limited power to statistically discern such differences.

### Plasma DHPG and norepinephrine

**Figure 2:**
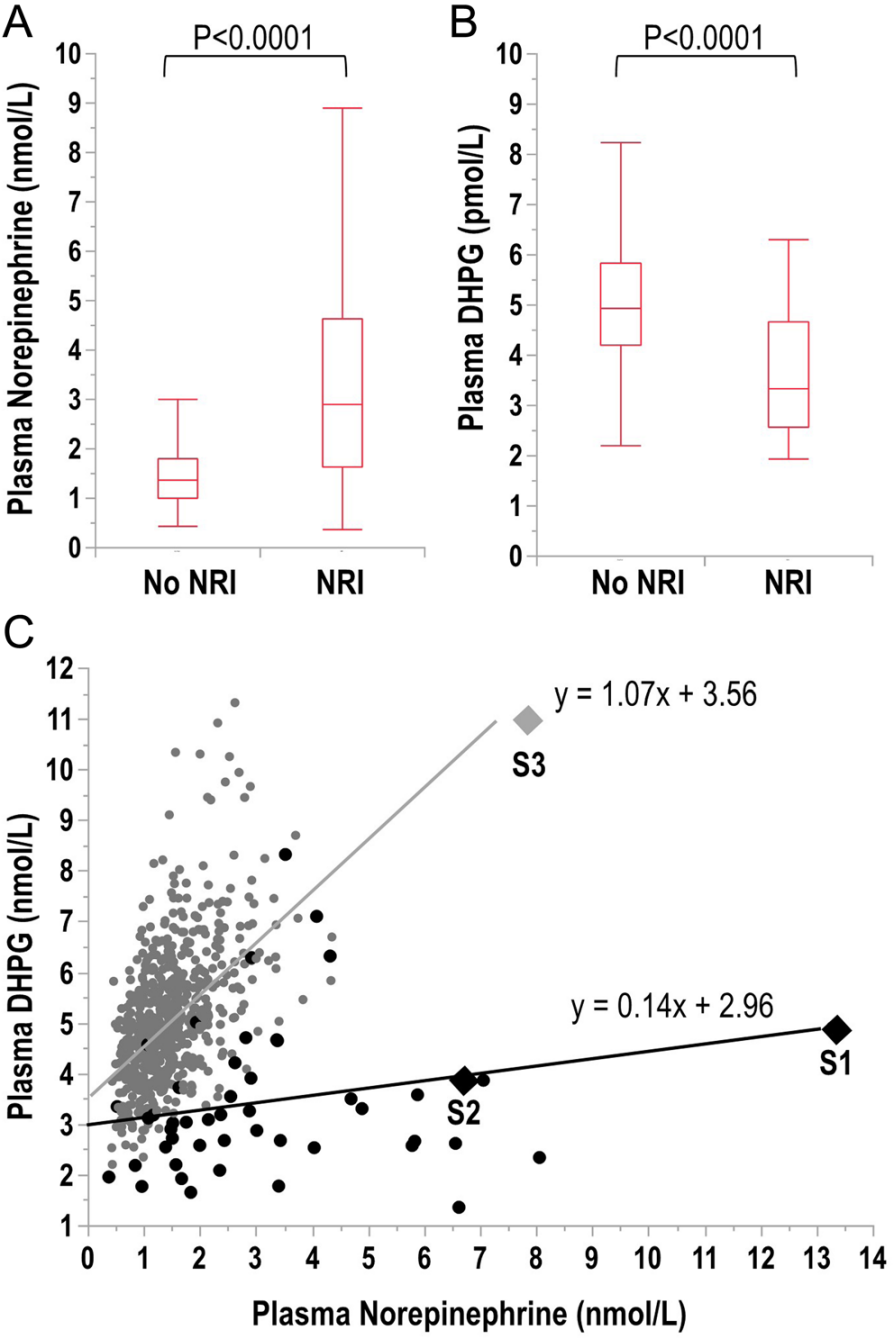
Box plot display of plasma norepinephrine (A) and DHPG (B) and relationships of plasma DHPG with norepinephrine (C) in patients with and without norepinephrine reuptake inhibition (NRI). In panel C, data for patients without NRI (gray dots) show a positive relationship (*r*_s_ = 0.515, *P* < 0.0001), whereas data for patients in whom norepinephrine reuptake was blocked (black dots) show a relationship (*r*_s_ = 0.284, *P* = 0.048) with an 87% decrease in the slope compared to patients with intact norepinephrine transporter function. Data are also shown for the three sampling time points (S1, S2, S3) in the patient of the case presentation (diamonds), the first two sampling points (S1 and S2) with and the third without the norepinephrine reuptake blockade. Box plots show medians and interquartile ranges.

To investigate the efficacy of norepinephrine reuptake inhibition, plasma norepinephrine and DHPG were measured in the presence and absence of NRIs. Patients treated with NRIs had 37% lower (*P* < 0.0001) plasma concentrations of DHPG and 113% higher (*P* < 0.0001) plasma concentrations of norepinephrine than in the normotensive and hypertensive volunteers in whom norepinephrine neuronal transporter function was intact (Fig. 2A and B). There were positive relationships between plasma norepinephrine and DHPG for patients in whom norepinephrine transporter function was intact (*r*_s_ = 0.515, *P* < 0.0001) and blocked by NRIs (*r*_s_ = 0.284, *P* = 0.0482), though the slope of relationships was reduced by 87% in patients taking NRIs (Fig. 2C).

### Case presentation

**Figure 3:**
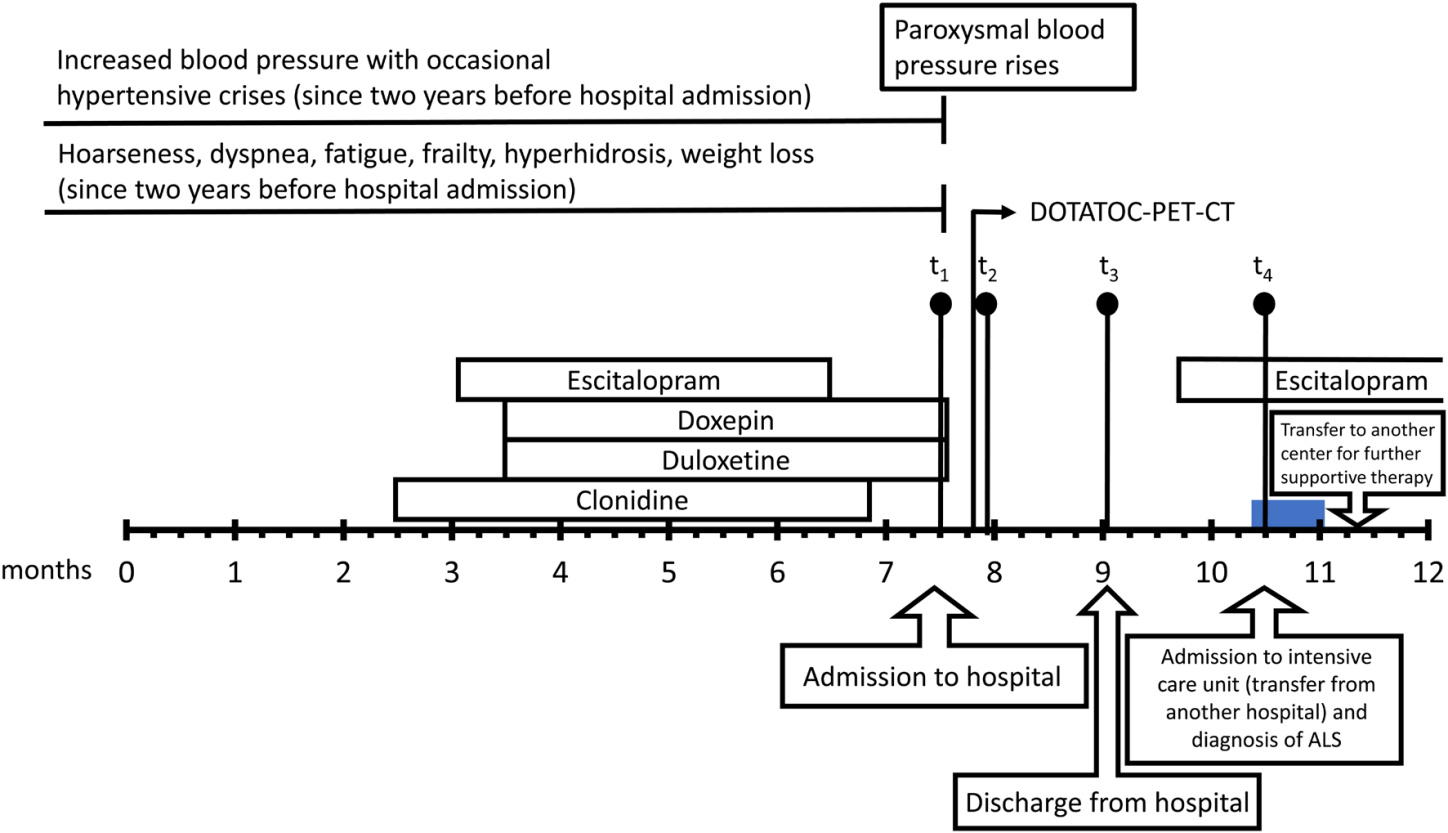
Timeline of medical history, including symptoms, relevant medications and measurements of metanephrines (t_1_, t_2_, t_3_, t_4_), for the patient chosen for the case presentation. The blue bar indicates time of artificial ventilation. Bars without coloring indicate times of medication administration.

A 74-year-old woman was admitted to the University Hospital of Leipzig with signs and symptoms that raised the suspicion of a PPGL (Fig. 3). The patient received candesartan and metoprolol because of hypertension. Treatment with doxepin (25 mg 1 × 1) and duloxetine (90 mg 1 × 1) due to recurrent depressive episodes was initiated roughly 4 months before admission, along with clonidine. One month before hospitalization, the dose (0.15 mg 4 times daily) of clonidine was tapered according to the most recent prescribing information until complete withdrawal 2 weeks before admission.

Due to hypertensive episodes and symptoms suggestive of a catecholamine-producing tumor, blood samples were drawn for measurements of plasma-free metanephrines and methoxytyramine two days after admission. Results showed plasma concentrations of normetanephrine and methoxytyramine 7.8- and 6.6-fold higher than upper cutoffs of reference intervals (Table 2). Compared to the study populations, these concentrations were above respective medians of patients with PPGL and well above the highest false-positive plasma normetanephrine concentration in patients without PPGL, including others taking NRIs (Fig. 1).

**Table 2:** Plasma concentrations of catecholamine metabolites and catechols at different time points in the presented patient.

	Reference intervals	Hospital admission	2 weeks later	7 weeks later	3 months later
*O*-methylated catecholamine metabolites					
Normetanephrine	0.17–1.04	8.15	4.71	2.04	0.42
Metanephrine	0.06–0.43	0.35	0.31	0.39	0.16
Methoxytyramine	0.01–0.10	0.66	0.14	0.07	0.04
Catechols					
Norepinephrine	0.57–3.03	13.29	7.06	7.82	
Epinephrine	0.02–0.45	0.26	0.31	0.34	
Dopamine	0.01–1.03	0.59	0.20	0.17	
Dihydroxyphenylalanine	4.98–12.92	16.4	14.9	21.2	
Dihydroxyphenylacetic acid	4.68–33.71	11.74	9.41	17.48	
DHPG	2.94-8.22	4.90	3.86	10.95	
DHPG–norepinephrine ratio	1.83-7.48	0.37	0.55	1.40	

Values are shown as nmol/L; 2 weeks, 7 weeks and 3 months refer to different time points after hospital admission. DHPG, dihydroxyphenylglycol.

Despite the highly elevated plasma concentrations of normetanephrine and methoxytyramine, somatostatin receptor imaging yielded no indication of an adrenal or extra-adrenal mass in this patient. Given the possibility of false-positive biochemical test results, doxepin and duloxetine were discontinued (Fig. 3). This resulted in a continued lowering of plasma concentrations of normetanephrine and methoxytyramine (Table 2).

Plasma catechols were analyzed to evaluate any associated derangements of sympathoneuronal function, and particularly the function of neuronal reuptake as assessed by measurements of DHPG and norepinephrine. The DHPG–norepinephrine ratio was 0.37 at time of admission and showed a steady rise to 1.43 at 7 weeks after cessation of doxepin and duloxetine. All other plasma catechols were within normal limits. As further shown in Fig. 2, plasma concentrations of DHPG and norepinephrine at the two initial sampling time points fell within relationships of other patients taking NRIs; at 7 weeks after discontinuation of NRIs, the plasma concentrations of norepinephrine remained elevated, though at a point in the relationship with DHPG that was consistent with normalized norepinephrine transporter function.

Three months post admission, the patient was rehospitalized due to respiratory insufficiency. Shortly thereafter, EMG/ENG findings finally led to the diagnosis of amyotrophic lateral sclerosis (ALS), which explained initial symptoms of fatigue, shortness of breath, hoarseness and weight loss. Remarkably, a blood collection in the ICU yielded entirely normal plasma concentrations of free metanephrines and methoxytyramine (Table 2).

## Discussion

This study confirms previous findings that TCAs but not SSRIs increase plasma concentrations and rates of false positives for normetanephrine (Eisenhofer *et al.* 2003); however, for the first time, the study also establishes similar actions on methoxytyramine and extends the findings for normetanephrine to patients taking SNRIs. Although the NRI-associated increases in plasma normetanephrine and methoxytyramine are usually mild, we also show that there can be exceptions where results for both normetanephrine and methoxytyramine extend well into the pathological range of patients with PPGLs. These observations are of clinical significance, since they clarify that when NRIs are associated with positive test results there should be considerable caution in interpreting those results even when highly elevated.

Activation of the sympathetic nervous system with sampling blood in the seated position is associated with a 30% increase in normetanephrine concentrations and a 2.8-fold increase in false-positive test results compared to sampling in the supine position (Lenders *et al.* 2007, de Jong *et al.* 2009). Additional nonadherence to an overnight fast further increases rates of false positives six-fold (Darr *et al.* 2014). These observations underline the importance of blood sampling in the supine position, and after an overnight fast when measuring methoxytyramine. On the other hand, withdrawal of NRIs is not recommended unless there is a positive test result, and then only after careful consideration of the need for mental health support.

Interestingly, TCAs resulted in a larger increase in plasma methoxytyramine than observed for SNRIs. As most patients were either on amitriptyline or on venlafaxine, one possible explanation for our results could be the higher affinity of amitriptyline for the dopamine transporter compared to venlafaxine (Tatsumi *et al.* 1997).

Plasma DHPG is partly derived from intraneuronal deamination of norepinephrine recaptured by sympathetic neurons (Goldstein *et al.* 1988). The metabolite thereby provides a biomarker of neuronal norepinephrine transporter function that can be used to assess actions of NRIs (Chappell *et al.* 2014). Thus, for example, the lower plasma concentrations of DHPG relative to norepinephrine in patients taking NRIs, as indicated by the 87% decrease in the slope of the relationship of DHPG with norepinephrine, reflect the extent of reuptake blockade.

**Figure 4:**
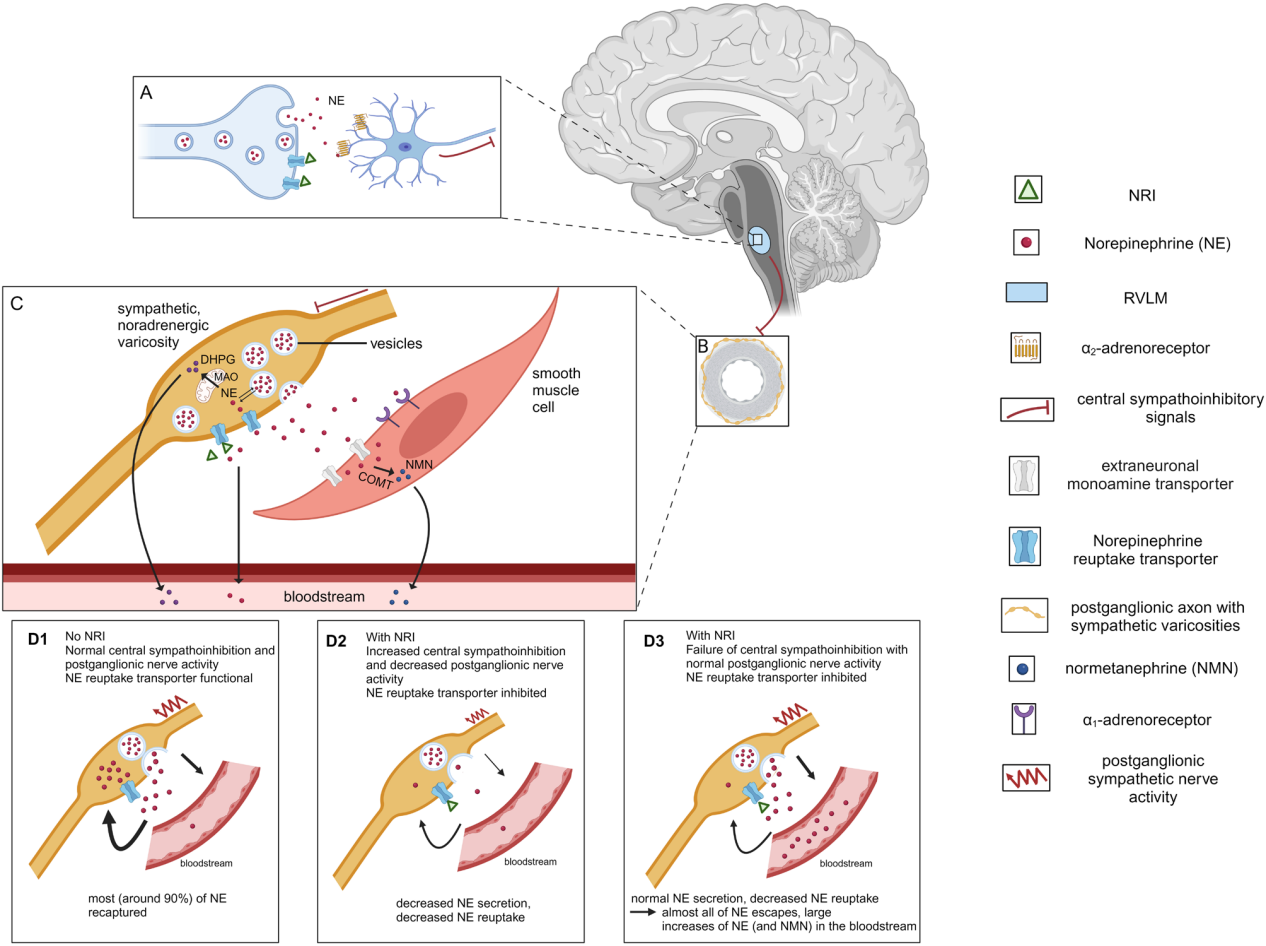
Concept of central and peripheral actions of norepinephrine reuptake inhibitors (NRIs) and relevant monoamine pathways. (A: Noradrenergic axonal terminal with postsynaptic α_2_-adrenoreceptors in the rostral ventrolateral medulla (RVLM). NRIs block the norepinephrine transporter, increasing local concentrations of catecholamines which then activate central α_2_-adrenoreceptors to increase sympathoinhibitory signals to postganglionic sympathetic neurons (C) in arterioles (B). (C) Enlarged view of a single sympathetic varicosity. Released norepinephrine is recaptured via norepinephrine transporters or metabolized extraneuronally to normetanephrine (NMN) by catechol-*O*-methyltransferase (COMT). Reuptake into the varicosity can sequester norepinephrine back into vesicles or deaminate it to dihydroxyphenylglycol (DHPG) via monoamine oxidase A (MAO). (D1–3) Zoom-in on sympathetic noradrenergic varicosities, illustrating norepinephrine levels in the bloodstream depending on NRI presence, central sympathoinhibition efficacy (and resulting postganglionic sympathetic nerve activity) and norepinephrine reuptake transporter functionality. In the presence of NRIs, central sympathoinhibition increases, leading to decreased postganglionic sympathetic nerve activity and norepinephrine release, but on the other hand, norepinephrine reuptake decreases, hence resulting in similar or slightly higher bloodstream norepinephrine levels (D2). With the norepinephrine reuptake blockade, but no increased sympathoinhibition during the presence of NRI, large proportions of norepinephrine escape to the bloodstream (D3). D3 represents the pathophysiology in the presented index patient. Created with BioRender.com

Since norepinephrine reuptake is the principal means for terminating the actions of the transmitter at sympathetic neuroeffector junctions (Axelrod & Kopin 1969), findings that acute administration of TCAs decrease rather than increase entry of norepinephrine into the circulation presented a paradox (Esler *et al.* 1981, 1991). This paradox was reinforced by the finding that close to 90% of all norepinephrine secreted by sympathetic nerves is removed by neuronal reuptake (Eisenhofer *et al.* 1991) so that blockade of the process would be expected to result in 10-fold elevations in plasma concentrations of the transmitter. The paradox was resolved after it was established that NRIs not only block norepinephrine reuptake but also exert a profound central sympatholytic action due to activation of α_2_-adrenoreceptors in the rostral ventral lateral medulla (Fig. 4) (Eisenhofer *et al.* 1991, Esler *et al.* 1991, Huangfu *et al.* 1995). The centrally mediated sympathoinhibition caused by NRIs reduces sympathoneural release of norepinephrine so that acute blockade of reuptake does not increase escape of norepinephrine from sites of release. Nevertheless, with chronic administration of TCAs, there can be a reversal of sympathoinhibition (Veith *et al.* 1994).

The considerations mentioned earlier explain why among the patients on NRIs there may occur increases in both plasma norepinephrine and normetanephrine, though of a relatively limited nature compared what might otherwise be expected without central sympathoinhibition. Possibly, variations in central α_2_-adrenoreceptor-mediated sympathoinhibition may explain variable responses of norepinephrine and normetanephrine to NRIs, including the exceptional case described here and another previously reported patient on the SNRI venlafaxine (Neary *et al.* 2011).

There are two possibilities for why there might have been a failure of the centrally mediated sympatholytic actions of NRIs in our patient. First, the patient had been taking the α_2_-adrenoreceptor agonist clonidine, a centrally acting sympatholytic that after prolonged administration can result in downregulation of central α_2_-adrenoreceptors and a rebound increase in sympathetic outflow after withdrawal of the drug (Reid *et al.* 1977, Planz *et al.* 1982, Karachalios *et al.* 2005). Nonetheless, in our patient, the maximum daily dose of clonidine was under that described to usually cause withdrawal symptoms (Houston 1981), and there was also appropriate tapering of the drug before testing for PPGL. Therefore, adverse reactions to the drug combination seem an unlikely cause of the extraordinary biochemical test results for our patient.

A more likely possibility for the highly elevated biochemical test results relates to the diagnosis of ALS, a neurodegenerative disease that not only impacts motor neurons but may include a multisystem presentation with usually subtle yet variable impacts on autonomic function (Piccione *et al.* 2015). Activation of the sympathetic nervous system accompanied by increases in plasma norepinephrine has been described in the disease (Ziegler *et al.* 1980, Yamashita *et al.* 1997), which in some patients may contribute to marked fluctuations in blood pressure (Shimizu *et al.* 1994). These impacts and the associated impaired sympathetic neurocirculatory responses appear to have a central nervous system origin (Oey *et al.* 2002, Karlsborg *et al.* 2003). It is therefore plausible that the markedly elevated biochemical test results resulted from interrupted sympathoinhibitory signals from the central nervous system. Considering the data for plasma DHPG and norepinephrine, the sympathetic activation clearly extended to after the patient was withdrawn from NRIs when the norepinephrine reuptake transporter function was restored.

## Conclusions

In summary, our data show that TCAs and SNRIs result in an up to five-fold increase in rates of false-positive results for plasma normetanephrine and that for TCAs this extends to methoxytyramine. While increases in plasma concentrations of normetanephrine or methoxytyramine are usually limited, in some patients they may reach pathological levels typical of patients with PPGL. In the exceptional case described here, the extraordinarily elevated biochemical test results likely reflected a failure of normal sympatholytic responses to NRIs, most likely secondary to the complications of ALS. Taken together, these results highlight the importance of paying attention to relevant medications and clinical conditions when interpreting positive test results for plasma measurements of metanephrines and methoxytyramine.

## Declaration of interest

All authors declare that there is no conflict of interest that could be perceived as prejudicing the impartiality of the study reported.

## Funding

This work was funded by the German Research Foundation (Deutsche Forschungsgemeinschafthttp://dx.doi.org/10.13039/501100001659, DFG) – through SFB 1052, project number: 209933838, CRC 1052/3, subproject C06 (A.T.). R.S., and therefore this work, was funded by the German Research Foundation (Deutsche Forschungsgemeinschafthttp://dx.doi.org/10.13039/501100001659, DFG); project number 493646873 – MD-LEICS. Further, R.S. was funded by a travel grant for the 66th Annual Conference of the Deutsche Gesellschaft für Endokrinologie (DGE) in Baden-Baden, Germany, and was awarded with the ‘Junior Researcher Award/Young Talent Award’ of the applied endocrinology section of the Deutsche Gesellschaft für Endokrinologie (DGE). This study in part received funding from the ‘Deutsche Forschungsgemeinschafthttp://dx.doi.org/10.13039/501100001659‘ (314061271-TRR/CRC 205-1/2) to C.P., M.P. and G.E. for measurements of metanephrines in the case series data.

## Author contribution statement

R.S., C.P., M.P., A.T. and G.E. provided, analyzed and interpreted the data. R.S., C.P., A.T. and G.E. drafted the manuscript. N.R., O.S., S.R., R.B. and B.S. reviewed and revised the manuscript. All authors approved the final version of the manuscript.
